# Risk factors for neurological symptoms in hyponatraemic patients: a retrospective cohort study

**DOI:** 10.1093/ckj/sfaf357

**Published:** 2025-11-19

**Authors:** Akira Nakamura, Takashin Nakayama, Tatsuhiko Azegami, Motoaki Komatsu, Kaori Hayashi

**Affiliations:** Division of Nephrology, Endocrinology, and Metabolism, Department of Internal Medicine, Keio University School of Medicine, Tokyo, Japan; Division of Nephrology, Endocrinology, and Metabolism, Department of Internal Medicine, Keio University School of Medicine, Tokyo, Japan; Division of Nephrology, Endocrinology, and Metabolism, Department of Internal Medicine, Keio University School of Medicine, Tokyo, Japan; Department of Nephrology, Tokyo Saiseikai Central Hospital, Tokyo, Japan; Division of Nephrology, Endocrinology, and Metabolism, Department of Internal Medicine, Keio University School of Medicine, Tokyo, Japan

**Keywords:** encephalopathy, hyponatraemia, neurological symptoms

## Abstract

**Background:**

Hyponatraemia is one of the most common electrolyte disorders. While hyponatraemia can cause severe neurological symptoms, its clinical predictors remain poorly defined. This study aimed to identify factors associated with neurological involvement in patients with severe hyponatraemia.

**Methods:**

This retrospective cohort study included patients with severe hyponatraemia (serum sodium level ≤120 mEq/L) at admission or during hospitalization between January 2014 and June 2024 in two tertiary centres. Neurological symptoms were defined as vomiting, deep somnolence, seizures, coma or cardiopulmonary arrest. Risk factors were evaluated using multivariable logistic regression analysis.

**Results:**

Among 834 patients included in the analysis, the median (interquartile range) age was 73 (62–82) years, 382 (46%) were female and the median serum sodium level was 118 (116–119) mEq/L. Neurological symptoms were observed in 221 (26%). Low serum potassium [<4.0 mEq/L; odds ratio (OR) 1.53; 95% confidence interval (CI) 1.05–2.23] was independently associated with an increased risk of neurological symptoms, together with low serum sodium (<115 mEq/L; OR 2.23; 95% CI 1.49–3.33). Compared with chronic onset, both acute onset (OR 3.92; 95% CI 2.36–6.51) and uncertain onset (OR 2.05; 95% CI 1.40–2.99) also showed significant association with their occurrence.

**Conclusion:**

Low serum potassium, low serum sodium and onset pattern were independent predictors of neurological symptoms in patients with severe hyponatraemia. Particularly in those with these risk factors, careful assessment and individualized management of sodium imbalance may be appropriate to achieve favourable outcomes.

KEY LEARNING POINTS **What was known:**

- Severe hyponatraemia can cause neurological symptoms, but the clinical factors predisposing to these manifestations were not clearly defined.

**This study adds:**

- In a retrospective cohort involving 834 patients with severe hyponatraemia, low serum potassium, low serum sodium and onset pattern were independently associated with neurological symptoms.
- Low serum potassium emerged as a novel determinant of neurological symptoms, whereas the previously suggested associations of low serum sodium and acute onset—supported only by limited evidence—were further substantiated in this study.

**Potential impact:**

- Identifying high-risk patients is required to guide thoughtful evaluation and management of sodium imbalance, thereby helping to optimize the rate of correction.

## INTRODUCTION

Hyponatraemia is one of the most common electrolyte abnormalities, affecting more than 20% of hospitalized patients worldwide [1–4]. In hypotonic hyponatraemia, the osmotic gradient drives water into the intracellular compartment, leading to cellular swelling. In addition to osmotic mechanisms, hyponatraemia alters neuronal excitability and network synchronization by disturbing the balance between excitatory and inhibitory neurotransmission [5–7]. Through these mechanisms, hyponatraemia induces neurological symptoms, which may in turn underlie the consistently poor clinical outcomes observed in affected patients [8–10].

Clinical manifestations of hyponatraemia vary considerably among patients, ranging from asymptomatic to life-threatening presentations such as seizures or coma [3, 4]. On the other hand, the clinical characteristics that predispose patients to neurological manifestations remain insufficiently understood [8, 11–18]. Although previous reports have indicated that neurological involvement tends to occur more often in patients with acute onset or lower sodium levels, the supporting evidence remains limited. Moreover, other potential determinants have rarely been investigated to date [11, 13, 17].

Against this background, we aimed to examine risk factors for the development of neurological symptoms in patients with severe hyponatraemia. A better understanding of these relationships could facilitate the identification of patients prone to such complications and contribute to optimizing the clinical management of hyponatraemia.

## MATERIALS AND METHODS

### Study population

We performed a retrospective cohort study including patients with severe hyponatraemia (serum sodium ≤120 mEq/L) [19, 20], diagnosed either at admission or during hospitalization, between January 2014 and June 2024 at Keio University Hospital and Tokyo Saiseikai Central Hospital. The exclusion criteria were as follows: (i) obvious measurement errors; (ii) age under 18 years; (iii) blood glucose >500 mg/dL; (iv) management without hospitalization; (v) absence of follow-up; and (vi) inability to report symptoms due to preexisting impairment of consciousness or cognition before the onset of hyponatraemia. In patients with multiple episodes of severe hyponatraemia, only the first episode was taken into account. This study protocol was reviewed and approved by the Ethics Committee of Keio University School of Medicine (approval number: 20 251 007) and conducted in accordance with the principles of the Declaration of Helsinki. Informed consent was secured through an opt-out procedure provided on the institutional website.

### Data collection

Electronic medical records were reviewed to collect information such as age, sex, height, weight, blood pressure, heart rate, clinical setting at the time of hyponatraemia onset (inpatient or outpatient), comorbidities and concurrent medications. The onset pattern of hyponatraemia was categorized as acute (≤48 h), chronic (>48 h) or uncertain, when the time of onset could not be determined.

Laboratory data at the onset of hyponatraemia were obtained, including serum sodium, potassium, urea nitrogen, creatinine, urine sodium and urine potassium. The estimated glomerular filtration rate was calculated using the following equation: 194 × Serum creatinine^−1.094^ × Age^−0.287^ (× 0.739 if female) [21].

### Outcomes

The primary objective of this study was to identify the risk factors for hyponatraemia-related symptoms, which were classified into three categories: none or mild (fatigue, dizziness), moderate (confusion, nausea without vomiting, headache) and severe (vomiting, deep somnolence, seizures, coma, cardiopulmonary arrest) [4, 12]. Symptoms were regarded as attributable to hyponatraemia only when present at low sodium levels and could not be accounted for by other underlying conditions.

### Statistical analyses

Baseline clinical characteristics and laboratory data were assessed across three severity groups. Continuous variables are presented as medians (interquartile range), and categorical variables as numbers (percentages). Differences in continuous variables among the three groups were assessed using the Kruskal–Wallis test. For categorical variables, differences were examined using the chi-square test.

Logistic regression analyses were conducted to examine risk factors for the development of neurological symptoms, defined as severe manifestations. Results are presented as odds ratios (ORs) with 95% confidence intervals (CIs). Based on clinical relevance and pathophysiological rationale, the following variables were included as covariates in the multivariable models: age, sex, body mass index (BMI), Charlson comorbidity index (CCI), onset pattern (acute, chronic or uncertain), serum sodium (<115 vs ≥115 mEq/L), serum potassium (<4.0 vs ≥4.0 mEq/L) and urea nitrogen [8, 12]. Multivariable analyses were performed using complete case analysis.

To ensure the robustness of our findings, we performed seven sensitivity analyses. First, we substituted severe symptoms with moderate-to-severe symptoms as the outcome. Second, we conducted a sensitivity analysis excluding patients who presented with vomiting as the sole symptom, as it may typically have a bidirectional relationship with hyponatraemia. Third, we replaced CCI with individual variables for stroke, dementia and schizophrenia. Fourth, we excluded patients with acute hyponatraemia to clarify risk factors independent of onset pattern. Fifth, we included the clinical setting of hyponatraemia (outpatient vs inpatient) as a covariate. Sixth, we substituted age and sex with a binary covariate indicating female under 50 years versus all other patients, given previous reports suggesting an increased risk of brain damage in premenopausal women (aged <50 years) [12, 14]. Seventh, missing BMI values were addressed using multiple imputation by chained equations with 20 iterations [22].

We carried out all analyses using Stata version 18.0 (StataCorp LLC, College Station, TX, USA). All *P*-values were two-sided, and *P*-values <.05 were considered statistically significant.

## RESULTS

### Characteristics of the study population

**Figure 1:**
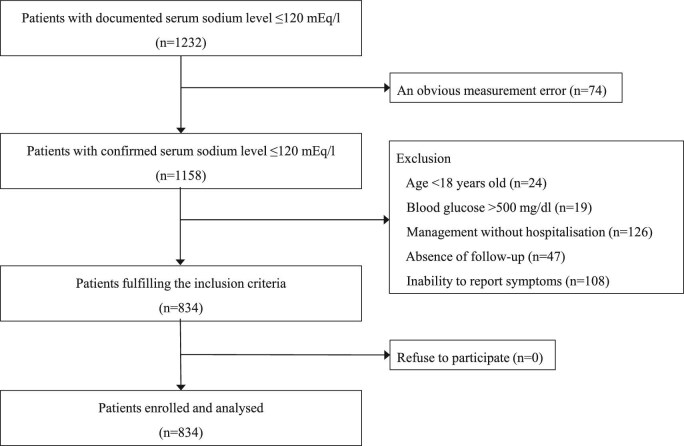
Flowchart of patient selection.

Among 1232 patients with serum sodium ≤120 mEq/L, 398 were excluded (Fig. 1): 74 had obvious measurement errors, 24 were under 18 years old, 19 had blood glucose levels >500 mg/dL, 126 were not admitted, 47 had no follow-up and 108 were unable to report symptoms prior to the onset of hyponatraemia. Consequently, 834 patients were included in the final analysis.

**Figure 2:**
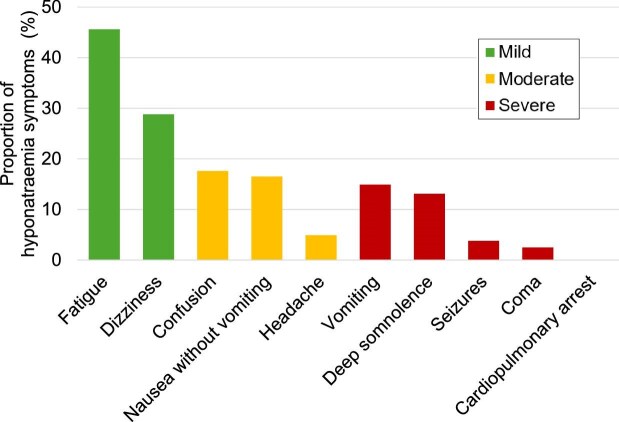
Symptoms in patients with severe hyponatraemia. Green bars indicate mild symptoms, yellow bars indicate moderate symptoms, and red bars indicate severe symptoms.

Baseline characteristics are described in Table 1. The median age was 73 (62–82) years, 382 (46%) were female and serum sodium was 118 (116–119) mEq/L. The distribution of symptoms is shown in Fig. 2. Patients were stratified into three groups according to symptom severity at the onset of hyponatraemia: 403 (48%) patients had no or mild symptoms—fatigue [380 (45.6%)] or dizziness [240 (28.8%)]; 210 (25%) had moderate symptoms—confusion [147 (17.6%)], nausea without vomiting [138 (16.5%)] or headache [41 (4.9%)]; and 221 (26%) had severe symptoms—vomiting [124 (14.9%)], deep somnolence [109 (13.1%)], seizures [32 (3.8%)] or coma [21 (2.5%)]. No patients experienced cardiopulmonary arrest. Patients with severe symptoms were more likely to be female, have an outpatient onset, exhibit an acute clinical course and show a lower CCI. Lower serum sodium, potassium and urea nitrogen levels were also observed in this group.

**Table 1:** Baseline patient characteristics by hyponatraemia-associated symptoms.

Variables	Overall (*n* = 834)	None/mild (*n* = 403)	Moderate (*n* = 210)	Severe (*n* = 221)	*P*-value
Age, years	73 (62–82)	73 (63–81)	73 (62–82)	73 (62–83)	.99
Female, *n* (%)	382 (46)	166 (41)	102 (49)	114 (52)	.03
Body mass index (*n* = 776)	20 (18–23)	20 (17–23)	20 (17–22)	20 (18–23)	.29
Outpatient status, *n* (%)	426 (51)	185 (46)	110 (52)	131 (59)	<.01
Onset patterns, *n* (%)					
Acute	100 (12)	20 (5)	34 (16)	46 (21)	<.01
Chronic	413 (50)	247 (61)	99 (47)	67 (30)	
Uncertain	321 (38)	136 (34)	77 (37)	108 (49)	
Vital signs					
Systolic BP, mmHg	127 (109–145)	122 (106–139)	132 (115–148)	132 (113–150)	<.01
Diastolic BP, mmHg	73 (62–84)	70 (59–81)	74 (65–85)	79 (67–89)	<.01
HR, bpm	82 (71–93)	83 (70–95)	81 (72–90)	84 (72–95)	.14
Comorbidities					
Hypertension, *n* (%)	378 (45)	194 (48)	93 (44)	91 (41)	.23
Diabetes mellitus, *n* (%)	211 (25)	124 (31)	41 (20)	46 (21)	<.01
Chronic kidney disease, *n* (%)	141 (17)	87 (22)	30 (14)	24 (11)	<.01
Heart failure, *n* (%)	198 (24)	110 (27)	44 (21)	44 (20)	.06
Cirrhosis, *n* (%)	77 (9)	52 (13)	13 (6)	12 (5)	<.01
Stroke, *n* (%)	83 (10)	41 (10)	17 (8)	25 (11)	.53
Dementia, *n* (%)	63 (8)	23 (6)	16 (8)	24 (11)	.07
Schizophrenia, *n* (%)	14 (2)	6 (1)	2 (1)	6 (3)	.33
CCI	3 (1–6)	3 (2–6)	2 (1–6)	2 (1–5)	<.01
Medications, *n* (%)					
Loop diuretics	165 (20)	109 (27)	29 (14)	27 (12)	<.01
Thiazide diuretics	60 (7)	30 (7)	14 (7)	16 (7)	.94
MRA	108 (13)	70 (17)	20 (10)	18 (8)	<.01
NSAIDs	133 (16)	51 (13)	41 (20)	41 (19)	.04
Opioids	99 (12)	42 (10)	27 (13)	30 (14)	.45
Antiepileptics	41 (5)	10 (2)	17 (8)	14 (6)	<.01
Antipsychotics	46 (6)	15 (4)	12 (6)	19 (9)	.04
Antidepressants	62 (7)	22 (5)	14 (7)	26 (12)	.01
Laboratory data					
Serum Na+, mEq/L	118 (116–119)	119 (117–120)	118 (115–119)	117 (114–119)	<.01
Serum K+, mEq/L	4.4 (3.9–5.0)	4.6 (4.1–5.0)	4.3 (3.8–4.7)	4.2 (3.7–4.9)	<.01
Serum UN, mg/dL	15 (10–26)	16 (11–30)	14 (10–22)	13 (9–24)	<.01
Serum Cr, mg/dL	0.70 (0.51–1.11)	0.77 (0.55–1.22)	0.65 (0.49–1.02)	0.62 (0.47–0.93)	<.01
eGFR, mL/min/1.73 m²	75 (46–104)	71 (39–99)	80 (48–102)	83 (54–113)	<.01
Urine Na^+^, mEq/L (*n* = 545)	57 (27–100)	55 (25–92)	60 (31–107)	59 (26–112)	.21
Urine K^+^, mEq/L (*n* = 530)	30 (20–43)	30 (17–41)	31 (24–45)	30 (21–43)	.24

Continuous variables are presented as medians (interquartile range), and categorical variables as numbers (percentages). BMI, body mass index; BP, blood pressure; HR, heart rate; CCI, Charlson comorbidity index; MRA, mineralocorticoid receptor antagonists; NSAIDs, nonsteroidal anti-inflammatory drugs; Na^+^, sodium; K^+^, potassium; UN, urea nitrogen; Cr, creatinine; eGFR, estimated glomerular filtration rate.

### Logistic regression analyses

As shown in Table 2, unadjusted logistic regression demonstrated that female, low CCI acute onset, uncertain onset, low serum sodium <115 mEq/L and low serum potassium <4.0 mEq/L were all related to higher risk of developing neurological symptoms. In the adjusted multivariable model, acute onset (OR 3.92; 95% CI 2.36–6.51) and uncertain onset (OR 2.05; 95% CI 1.40–2.99), low serum sodium (OR 2.23; 95% CI 1.49–3.33) and low serum potassium (OR 1.53; 95% CI 1.05–2.23) remained independently associated with the outcomes.

**Table 2:** Results of logistic regression analysis for neurological symptoms.

	OR (95% CI)	
	Unadjusted model	Multivariate model
Age (per 10 years)	0.97 (0.87–1.08)	1.03 (0.92–1.16)
Female	1.37 (1.01–1.87)	1.16 (0.82–1.64)
Body mass index	1.02 (0.98–1.06)	1.03 (0.99–1.07)
CCI	0.91 (0.86–0.97)	0.93 (0.87–1.00)
Onset patterns (vs chronic)		
Acute onset	4.40 (2.74–7.05)	3.92 (2.36–6.51)
Uncertain onset	2.62 (1.85–3.71)	2.05 (1.40–2.99)
Serum Na^+^ <115 mEq/L	2.47 (1.72–3.56)	2.23 (1.49–3.33)
Serum K^+^ <4.0 mEq/L	1.97 (1.41–2.75)	1.53 (1.05–2.23)
Serum UN (per 10 mg/dL)	0.99 (0.93–1.05)	1.05 (0.98–1.12)

Na^+^, sodium; K^+^, potassium; UN, urea nitrogen.

Seven sensitivity analyses were conducted. First, when moderate-to-severe symptoms were used as the outcome, the four risk factors remained significant (Supplementary data, Table S1). Second, in the analysis excluding 96 patients with vomiting as the only symptom, findings were largely in agreement with the main results (Supplementary data, Table S2). Third, replacing CCI with stroke, dementia and schizophrenia as covariates did not materially affect the results (Supplementary data, Table S3). Fourth, exclusion of patients with acute hyponatraemia yielded consistent results (Supplementary data, Table S4). Fifth, inclusion of the clinical setting at hyponatraemia onset as a covariate did not change the associations (Supplementary data, Table S5). Sixth, substitution of age and sex with a binary variable for female under 50 years yielded consistent results (Supplementary data, Table S6). Seventh, the sensitivity analysis using multiple imputation for missing BMI values also produced results consistent with the primary analysis (Supplementary data, Table S7).

## DISCUSSION

The present study analysed approximately 800 patients with serum sodium ≤120 mEq/L to identify risk factors for neurological symptoms associated with severe hyponatraemia. This represents one of the largest cohorts examined to date and allowed robust multivariable logistic regression and multiple sensitivity analyses. We confirmed previously suggested associations of acute onset and low serum sodium, and importantly, demonstrated for the first time that low serum potassium independently increases the risk of neurological involvement.

Neurological manifestations of hyponatraemia have been well described in observational series [8, 13, 18]; however, few studies have systematically examined the risk factors for their occurrence [11, 17]. Both onset pattern and sodium levels have been implicated as contributing factors. In a cohort of 66 patients with serum sodium concentrations <128 mmol/L, Arieff *et al*. reported that acute hyponatraemia was associated with markedly higher rates of stupor, coma or seizures compared with chronic cases [11, 17]. Lower serum sodium within the same cohort was linked to an increased likelihood of neurological complications. However, given the small sample size and lack of adjusted analyses limited the robustness of these earlier findings, underscoring the need for larger, methodologically rigorous studies.

To our knowledge, this is the first large-scale study to comprehensively evaluate the risk factors for neurological symptoms in hyponatraemia using multivariable logistic regression analyses. Consistent with previous indications, acute onset was significantly associated with the presence of such manifestations compared with chronic onset. Patients with uncertain onset showed a similar but less pronounced trend, likely reflecting a mixture of acute and chronic cases [11, 17]. As anticipated, lower serum sodium also showed a significant relationship with symptom presentation, whereas lower serum potassium independently predicted neurological involvement—a previously unrecognized finding [8, 11, 13, 18].

Interestingly, female sex was not significantly associated with hyponatraemia-related symptoms in this cohort. In contrast, postoperative hyponatraemia accompanied by neurological involvement has been predominantly reported in women [12, 16]. Although speculative, this discrepancy may be attributable to differences in patient characteristics, including ethnicity, as well as to the use of multivariable adjustment in the present analysis. Further studies are warranted to evaluate whether sex-related differences genuinely exist in the clinical manifestations of hyponatraemia.

The observed associations may be accounted for by several mechanistic pathways. In acute hyponatraemia, insufficient time for cerebral osmotic adaptation leads to abrupt intracellular water influx and neuronal swelling [11, 12, 17, 23]. In contrast, chronic hyponatraemia allows gradual osmolyte efflux, which helps mitigate cerebral water accumulation and reduces the likelihood of clinically overt neurological dysfunction. Lower serum sodium concentrations may further augment the transmembrane osmotic gradient, thereby exacerbating cellular oedema and predisposing to symptomatic presentation [11]. In addition, our study identified an association between potassium levels and neurological symptoms, possibly reflecting a role of potassium in neurological vulnerability in this context. Na⁺–K⁺–ATPase activity is essential for cell-volume regulation during early adaptation to hypotonic stress [24, 25], and impairment of its function under hypokalaemic conditions potentially limits osmotic efflux of intracellular water and promotes cellular swelling [26]. It is worth noting that neurological symptoms in hyponatraemia may involve mechanisms beyond cerebral oedema, with neurotransmitter-mediated effects (e.g. glutamate release) reported to play an important role [6, 7]. The underlying pathophysiology remains unclear, underscoring the need for further investigation.

Our findings have important clinical implications. The nonspecific nature of hyponatraemia manifestations makes accurate clinical evaluation difficult, thereby resulting in missed or delayed recognition. Although timely correction is desirable in symptomatic cases, clinicians often err on the side of caution because of concerns about osmotic demyelination syndrome [3, 4, 27]. In this study, we identified factors associated with an increased likelihood of hyponatraemia-related neurological symptoms, which may help improve the quality of patient assessment and promote timely therapeutic intervention when indicated. Furthermore, the prevalence of chronic kidney disease and heart failure—conditions that predispose to disturbances of water and electrolyte homeostasis—has been rising in recent years [10]. In addition to being prone to hyponatraemia, patients with these conditions often experience potassium abnormalities, including hyperkalaemia and hypokalaemia, due to the use of renin–angiotensin system inhibitors and potassium-wasting diuretics [28]. Our findings, demonstrating a link between potassium levels and symptom presentation, underscore the importance of careful attention to potassium balance in the management of hyponatraemia.

This study has several limitations that should be acknowledged. Because of its retrospective design, the direction of associations cannot be determined, and residual confounding and documentation bias cannot be excluded despite adjustment with multivariable analyses. The lack of objective intracranial data such as head computed tomography or magnetic resonance imaging represents a major constraint. Neurological assessment therefore relied on medical record review, which may have introduced inaccuracy and a risk of misclassification. We regarded symptoms that were present at the time of, but not before or after, the episode as attributable to hyponatraemia, although the involvement of other pathological conditions could not be entirely ruled out. It is noteworthy that approximately half of the patients in the present study presented with pronounced symptoms beyond fatigue or dizziness, which were largely consistent with previous reports [1, 8, 13, 18]. The exclusion of patients unable to report symptoms because of preexisting neurological impairment could have contributed to selection bias. As this study was conducted in tertiary care hospitals in Japan, the generalizability of the findings to other healthcare settings may be limited. Moreover, we did not evaluate whether the presence of neurological symptoms in hyponatraemia was related to subsequent clinical outcomes, which remains an important question for future investigation. Given the limited sample size, the present study was unable to examine individual neurological manifestations in detail, and the analysis was instead performed collectively according to classifications described in the previous literature [4]. It should be noted, however, that these classifications are not universally established and may differ across guidelines [3, 4]. In particular, vomiting—being both a cause and a consequence of hyponatraemia—warrants attention. The potential impact of this condition on potassium balance indicates that the significance of the observed potassium levels should be interpreted with caution. These limitations highlight the need for larger, prospective investigations.

In conclusion, this study demonstrated that low serum potassium, along with onset pattern and low serum sodium, was significantly associated with an increased risk of developing neurological symptoms in patients with severe hyponatraemia. Awareness of these risk factors in clinical settings may support appropriate assessment and management of hyponatraemia.

## Supplementary Material

sfaf357_Supplemental_File

## Data Availability

The data underlying this article will be shared on reasonable request to the corresponding author.
