# Supplementary material for: Risk factors for neurological symptoms in hyponatraemic patients: a retrospective cohort study
Source: Clin Kidney J. 2025 Nov 19;18(12):sfaf357. doi: 10.1093/ckj/sfaf357 (PMC12690194; doi:10.1093/ckj/sfaf357)
Supplement: sfaf357_Supplemental_File [file sfaf357_supplemental_file.docx]

**Supplemental Material**

***Risk Factors for* Neurological *Symptoms in Hyponatraemic Patients: A Retrospective Cohort Study***

Akira Nakamura^1^*, Takashin Nakayama^1^*, Tatsuhiko Azegami^1^, Motoaki Komatsu^2^, Kaori Hayashi^1^

*These authors contributed equally to this work.

*1 Division of Nephrology, Endocrinology, and Metabolism, Department of Internal Medicine, Keio University School of Medicine, Tokyo, Japan.*

*2 Department of Nephrology, Tokyo Saiseikai Central Hospital, Tokyo, Japan.*

**Table of contents**

**Supplementary Table 1. Results of logistic regression analysis for neurological symptoms (defined as moderate to severe, rather than severe only)**

**Supplementary Table 2. Results of logistic regression analysis for neurological symptoms (excluding patients who presented with vomiting as the sole symptom)**

**Supplementary Table 3. Results of logistic regression analysis for neurological symptoms (replacing Charlson comorbidity index with stroke, dementia and schizophrenia as covariates)**

**Supplementary Table 4. Results of logistic regression analysis for neurological symptoms (excluding patients with an acute clinical course)**

**Supplementary Table 5. Results of logistic regression analysis for neurological symptoms (incorporating clinical setting as an additional covariate)**

**Supplementary Table 6. Results of logistic regression analysis for neurological symptoms (replacing age and sex with female under age 50 as a covariate)**

**Supplementary Table 7. Results of logistic regression analysis for neurological symptoms (using multiple imputation)**

| **Supplementary Table 1. Results of logistic regression analysis for neurological symptoms (defined as moderate to severe, rather than severe only)** | | | |
| --- | --- | --- | --- |
|  | | Odds ratio (95% confidence interval) | |
|  | | Unadjusted Model | Multivariate Model |
| Age (per 10 years) | | 0.97 (0.89─1.07) | 1.03 (0.93─1.15) |
| Female | | 1.43 (1.09─1.89) | 1.14 (0.83─1.56) |
| Body mass index | | 1.01 (0.98─1.05) | 1.02 (0.98─1.05) |
| Charlson comorbidity index | | 0.89 (0.84─0.94) | 0.91 (0.85─0.96) |
| Onset patterns (vs Chronic) | |  |  |
|  | ─Acute onset | 5.95 (3.51─10.09) | 5.22 (2.99─9.12) |
|  | ─Uncertain onset | 2.02 (1.51─2.72) | 1.45 (1.04─2.01) |
| Serum Na+ <115 mEq/l | | 2.97 (2.03─4.36) | 2.84 (1.87─4.30) |
| Serum K+ <4.0 mEq/l | | 2.10 (1.53─2.89) | 1.75 (1.22─2.52) |
| Serum UN (per 10 mg/dl) | | 0.97 (0.92─1.02) | 1.02 (0.96─1.09) |
| Abbreviations: Na+, sodium; K+, potassium; UN, urea nitrogen. | | | |

| **Supplementary Table 2. Results of logistic regression analysis for neurological symptoms (excluding patients who presented with vomiting as the sole symptom)** | | | |
| --- | --- | --- | --- |
|  | | Odds ratio (95% confidence interval) | |
|  | | Unadjusted Model | Multivariate Model |
| Age (per 10 years) | | 1.04 (0.92─1.19) | 1.18 (1.02─1.37) |
| Female | | 1.17 (0.80─1.69) | 0.97 (0.64─1.49) |
| Body mass index | | 1.02 (0.98─1.07) | 1.03 (0.98─1.08) |
| Charlson comorbidity index | | 0.89 (0.83─0.96) | 0.92 (0.84─1.00) |
| Onset patterns (vs Chronic) | |  |  |
|  | ─Acute onset | 4.64 (2.65─8.11) | 3.70 (2.03─6.73) |
|  | ─Uncertain onset | 2.65 (1.73─4.07) | 1.88 (1.17─3.01) |
| Serum Na+ <115 mEq/l | | 2.36 (1.52─3.65) | 2.04 (1.25─3.33) |
| Serum K+ <4.0 mEq/l | | 1.86 (1.25─2.77) | 1.59 (1.01─2.50) |
| Serum UN (per 10 mg/dl) | | 0.92 (0.84─1.01) | 0.98 (0.88─1.08) |
| Abbreviations: Na+, sodium; K+, potassium; UN, urea nitrogen. | | | |

| **Supplementary Table 3. Results of logistic regression analysis for neurological symptoms (replacing Charlson comorbidity index with stroke, dementia and schizophrenia as covariates)** | | | |
| --- | --- | --- | --- |
|  | | Odds ratio (95% confidence interval) | |
|  | | Unadjusted Model | Multivariate Model |
| Age (per 10 years) | | 0.97 (0.87─1.08) | 0.98 (0.87─1.11) |
| Female | | 1.37 (1.01─1.87) | 1.21 (0.85─1.71) |
| Body mass index | | 1.02 (0.98─1.06) | 1.03 (0.99─1.07) |
| Stroke | | 1.22 (0.74─2.01) | 1.17 (0.66─2.08) |
| Dementia | | 1.79 (1.05─3.06) | 2.02 (1.06─3.83) |
| Schizophrenia | | 2.11 (0.72─6.15) | 1.28 (0.34─4.81) |
| Onset patterns (vs Chronic) | |  |  |
|  | ─Acute onset | 4.40 (2.74─7.05) | 3.99 (2.40─6.63) |
|  | ─Uncertain onset | 2.62 (1.85─3.71) | 2.08 (1.42─3.03) |
| Serum Na+ <115 mEq/l | | 2.47 (1.72─3.56) | 2.16 (1.44─3.24) |
| Serum K+ <4.0 mEq/l | | 1.97 (1.41─2.75) | 1.54 (1.05─2.25) |
| Serum UN (per 10 mg/dl) | | 0.99 (0.93─1.05) | 1.03 (0.97─1.11) |
| Abbreviations: Na+, sodium; K+, potassium; UN, urea nitrogen. | | | |

| **Supplementary Table 4. Results of logistic regression analysis for neurological symptoms (excluding patients with an acute clinical course)** | | |
| --- | --- | --- |
|  | Odds ratio (95% confidence interval) | |
|  | Unadjusted Model | Multivariate Model |
| Age (per 10 years) | 0.96 (0.85─1.07) | 1.01 (0.89─1.15) |
| Female | 1.27 (0.90─1.78) | 1.16 (0.79─1.70) |
| Body mass index | 1.02 (0.98─1.07) | 1.03 (0.99─1.08) |
| Charlson comorbidity index | 0.91 (0.85─0.97) | 0.90 (0.83─0.97) |
| Serum Na+ <115 mEq/l | 2.88 (1.95─4.27) | 2.81 (1.85─4.28) |
| Serum K+ <4.0 mEq/l | 1.82 (1.26─2.64) | 1.56 (1.03─2.38) |
| Serum UN (per 10 mg/dl) | 1.01 (0.95─1.08) | 1.05 (0.98─1.12) |
| Abbreviations: Na+, sodium; K+, potassium; UN, urea nitrogen. | | |

| **Supplementary Table 5. Results of logistic regression analysis for neurological symptoms (incorporating clinical setting as an additional covariate)** | | | |
| --- | --- | --- | --- |
|  | | Odds ratio (95% confidence interval) | |
|  | | Unadjusted Model | Multivariate Model |
| Age (per 10 years) | | 0.97 (0.87─1.08) | 1.03 (0.92─1.16) |
| Female | | 1.37 (1.01─1.87) | 1.15 (0.81─1.64) |
| Body mass index | | 1.02 (0.98─1.06) | 1.03 (0.99─1.07) |
| Charlson comorbidity index | | 0.91 (0.86─0.97) | 0.93 (0.87─1.00) |
| Onset patterns (vs Chronic) | |  |  |
|  | ─Acute onset | 4.40 (2.74─7.05) | 4.03 (2.40─6.78) |
|  | ─Uncertain onset | 2.62 (1.85─3.71) | 1.95 (1.28─2.96) |
| Serum Na+ <115 mEq/l | | 2.47 (1.72─3.56) | 2.19 (1.45─3.29) |
| Serum K+ <4.0 mEq/l | | 1.97 (1.41─2.75) | 1.52 (1.04─2.22) |
| Serum UN (per 10 mg/dl) | | 0.99 (0.93─1.05) | 1.05 (0.98─1.12) |
| Outpatient status | | 1.57 (1.15─2.14) | 1.12 (0.74─1.72) |
| Abbreviations: Na+, sodium; K+, potassium; UN, urea nitrogen. | | | |

| **Supplementary Table 6. Results of logistic regression analysis for neurological symptoms (replacing age and sex with female under age 50 as a covariate)** | | | |
| --- | --- | --- | --- |
|  | | Odds ratio (95% confidence interval) | |
|  | | Unadjusted Model | Multivariate Model |
| Female under age 50 | | 1.87 (0.96─3.65) | 1.52 (0.73─3.17) |
| Body mass index | | 1.02 (0.98─1.06) | 1.03 (0.99─1.07) |
| Charlson comorbidity index | | 0.91 (0.86─0.97) | 0.94 (0.87─1.00) |
| Onset patterns (vs Chronic) | |  |  |
|  | ─Acute onset | 4.40 (2.74─7.05) | 3.93 (2.37─6.53) |
|  | ─Uncertain onset | 2.62 (1.85─3.71) | 2.06 (1.40─3.01) |
| Serum Na+ <115 mEq/l | | 2.47 (1.72─3.56) | 2.24 (1.50─3.35) |
| Serum K+ <4.0 mEq/l | | 1.97 (1.41─2.75) | 1.53 (1.05─2.21) |
| Serum UN (per 10 mg/dl) | | 0.99 (0.93─1.05) | 1.05 (0.98─1.12) |
| Abbreviations: Na+, sodium; K+, potassium; UN, urea nitrogen. | | | |

| **Supplementary Table 7. Results of logistic regression analysis for neurological symptoms (using multiple imputation)** | | | |
| --- | --- | --- | --- |
|  | | Odds ratio (95% confidence interval) | |
|  | | Unadjusted Model | Multivariate Model |
| Age (per 10 years) | | 0.97 (0.87─1.08) | 1.02 (0.91─1.13) |
| Female | | 1.37 (1.01─1.87) | 1.12 (0.80─1.57) |
| Body mass index | | 1.02 (0.99─1.06) | 1.03 (0.99─1.08) |
| Charlson comorbidity index | | 0.91 (0.86─0.97) | 0.95 (0.89─1.01) |
| Onset patterns (vs Chronic) | |  |  |
|  | ─Acute onset | 4.40 (2.74─7.05) | 4.17 (2.54─6.85) |
|  | ─Uncertain onset | 2.62 (1.85─3.71) | 2.21 (1.53─3.18) |
| Serum Na+ <115 mEq/l | | 2.47 (1.72─3.56) | 2.23 (1.52─3.28) |
| Serum K+ <4.0 mEq/l | | 1.97 (1.41─2.75) | 1.52 (1.11─2.28) |
| Serum UN (per 10 mg/dl) | | 0.99 (0.93─1.05) | 1.05 (0.98─1.13) |
| Abbreviations: Na+, sodium; K+, potassium; UN, urea nitrogen. Body mass index was imputed using multiple imputation. | | | |
